# Rapid resolution of acute subdural hematoma in child with severe head injury: a case report

**DOI:** 10.1186/1752-1947-7-67

**Published:** 2013-03-14

**Authors:** Jae-Young Park, Kyung-Sub Moon, Jung-Kil Lee, Kyung-Woon Jeung

**Affiliations:** 1 Department of Neurosurgery, Chonnam National University Hwasun Hospital, Hwasun-gun, Jeollanamdo 519-763, South Korea; 2 Department of Neurosurgery, Chonnam National University Hospital, Gwangju, South Korea; 3 Department of Emergency Medicine, Chonnam National University Hwasun Hospital, Hwasun-gun, Jeollanamdo, South Korea

**Keywords:** Acute subdural hematoma, Pediatric head injury, Rapid resolution, Spontaneous resolution

## Abstract

**Introduction:**

Rapid spontaneous resolution of traumatic acute subdural hematoma is an infrequent phenomenon and mainly develops in a case of simple acute subdural hematoma without parenchymal contusion. However, it has been rarely reported in a pediatric case with severe initial head injury.

**Case presentation:**

A 7-year-old Asian girl with traumatic acute subdural hematoma was transferred to our hospital for an emergency operation based on the results of an initial computed tomography scan and neurological examination. However, a repeat computed tomography scan two hours after trauma disclosed considerable reduction of the hematoma and midline shift with neurological improvements. Serial follow-up imaging studies demonstrated apparent redistribution of the hematoma over the cerebellar tentorium, posterior interhemispheric fissure and subarachnoid space. The patient was discharged with mild confusion 40 days after the admission.

**Conclusion:**

A follow-up computed tomography scan is strongly recommended before surgery when a child with a severe head injury presents with any sign of neurological improvement, especially with a mixed density hematoma on the initial computed tomography scan.

## Introduction

Traumatic acute subdural hematoma (ASDH) is a neurosurgical emergency with mortality as high as 60% to 80% [1]. Most of these patients undergo urgent hematoma evacuation via a craniotomy except for those with poor general condition or with irreversible brain damage. Several criteria have been used for the proper indication and timing of surgical evacuation for ASDH. In general, prompt surgical management of an ASDH with a thickness greater than 10mm or midline shift greater than 5mm on a computed tomography (CT) scan could be tried regardless of the patient’s Glasgow Coma Scale (GCS) score [2]. The clinical aspects are also important parameters for choosing the treatment options, in addition to the radiological criteria. Even in a comatose patient (GCS score less than nine) who has a hematoma that is less than 10mm thick and a midline shift less than 5mm, surgical evacuation should be performed if the patient’s GCS score decreases by two or more points during the hospitalization, or the patient presents with asymmetric or fixed and dilated pupils and/or the intracranial pressure (ICP) exceeds 20mmHg [3]. Even in a case not indicated for emergency operation, careful monitoring of the neurological status is mandatory because of the possibility of unexpected worsening of the neurological status.

By contrast, spontaneous resolution of an ASDH that initially required an operation is an infrequent phenomenon and mainly develops in a case of simple ASDH without parenchymal contusion [4-12]. To the best of our knowledge, spontaneous resolution of an ASDH has been rarely reported in a pediatric case [5,9,10], especially with a severe initial head injury. We present the clearly relevant images of such a case and discuss the mechanisms related to the rapid resolution of ASDH.

## Case presentation

**Figure 1:**
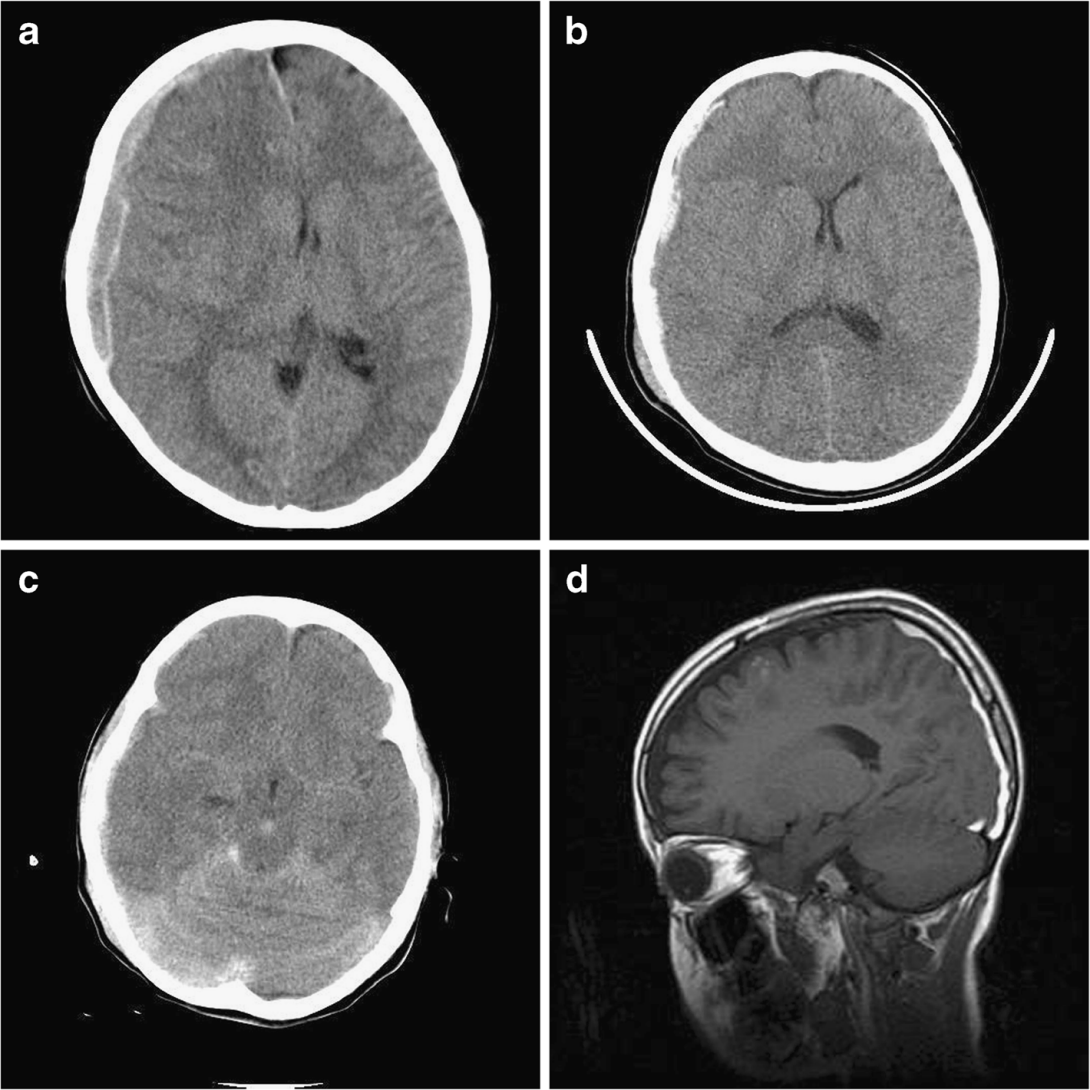
**a: Initial computed tomography scan demonstrating mixed density of right hemispheric acute subdural hematoma with midline shift of more than 10mm. b**: Computed tomography (CT) scan two hours after the accident showing marked reduction of the hematoma with more condensed density and improvement of the midline shift. **c**: CT scans six hours after the accident showing prominent subarachnoid hemorrhage and redistribution of the hematoma into the tentorial and interhemispheric subdural spaces. **d**: Sagittal T_1_-weighted magnetic resonance image, obtained two weeks after the accident, revealing a subacute-staged subdural hematoma with frontal contusion.

A 7-year-old Asian girl presented with deterioration of mental status to a nearby hospital after a pedestrian traffic accident (3:30 p.m.). The patient was comatose with a score of five on the GCS and had a left hemiparesis with a dilated right pupil. The CT scan, performed at 3:53 p.m., demonstrated a right frontotemporoparietal ASDH with severe midline shift (Figure 1a). The patient was transferred to our hospital for emergent surgery. A neurological examination revealed a score of 7T (eye=three, verbal=intubated, motor=four) on GCS without motor deficits or pupillary abnormalities. A repeat CT scan at 5:46 p.m. disclosed considerable reduction of the hematoma and midline shift (Figure 1b). Based on the definitive improvements of neurological signs and CT findings, we decided to monitor the neurological status and check the follow-up CT scan, with conservative medical treatment. A follow-up CT scan six hours after the accident showed a further resolution of the ASDH with apparent redistribution over the cerebellar tentorium, posterior interhemispheric fissure and subarachnoid space (Figure 1c). Magnetic resonance imaging (MRI), performed two weeks later, demonstrated a residual subacute subdural hematoma and multiple cerebral contusions (Figure 1d). The patient recovered consciousness on the tenth day and was discharged with mild confusion 40 days after the admission.

## Discussion

Even though traumatic ASDH is a neurosurgical emergency requiring immediate therapeutic intervention, some reports have shown unexpected resolution of the hematoma within two hours after the initial injury, which prevented the need for emergency surgery [4,6,8]. Rapid spontaneous resolution generally develops in a case of simple ASDH without parenchymal contusion, after a minor head injury [4,6].

Two possible mechanisms have been proposed for this infrequent phenomenon. First, the hematoma may be diluted by flow of cerebrospinal fluid (CSF) through the arachnoid tear, followed by retrograde flow into the subarachnoid space. This is supported by the presence of a low-density band between the ASDH and the inner table of the skull on CT scan [11]. Transient neurological deterioration with subsequent dramatic improvement may be related to CSF influx and efflux within the subdural space [4,6,8,11]. The prominent subarachnoid space with cerebral atrophy may facilitate dilution of ASDH [11]. Second, the compression and redistribution of the hematoma can be induced by cerebral swelling and increased ICP. This hypothesis is supported by the finding of dispersal of blood in the cerebellar tentorium or interhemispheric subdural space on the follow-up MRI [8,11]. In addition, the accompanying skull fracture and dural injury may allow the hematoma to drain into the subgaleal or intradiploic space [4]. Of interest, the reported cases of spontaneous resolution of ASDH generally occurred in aged patients. This reason can be explained by unique characteristics of the pediatric brain; there is no brain atrophy or excessive subarachnoid space to dilute or redistribute the hematoma.

In our case, marked hematoma reduction with condensed density and increased subarachnoid hemorrhage, on serial CT scans, can be explained by the dilution mechanism. Furthermore, the thickened tentorial and interhemispheric hematoma on follow-up imaging studies also support redistribution of the hematoma.

## Conclusion

Our case suggests that both dilution and redistribution of a hematoma account for the infrequent phenomenon of spontaneous resolution of an ASDH. Even in a child with a severe head injury, when there is any sign of neurological improvement, especially with a mixed density hematoma on the initial CT scan, a follow-up CT scan is strongly recommended before surgery.

## Consent

Written informed consent was obtained from the patient’s legal guardian for publication of this manuscript and accompanying images. A copy of the written consent is available for review by the Editor-in-Chief of this journal.

## Competing interests

The authors declare that they have no competing interests.

## Authors’ contributions

JYP wrote the manuscript. KSM performed the treatment and was the coordinator of the study. JKL summarized the patient notes and carried out the literature search. KWJ participated in the draft of the study, and in the conception of the study. All authors read and approved the final manuscript.
